# Nature of coexisting thyroid autoimmune disease determines success or failure of tumor immunity in thyroid cancer

**DOI:** 10.1186/s40425-018-0483-y

**Published:** 2019-01-07

**Authors:** Shahnawaz Imam, Pervaiz Dar, Rodis Paparodis, Khalil Almotah, Ahmed Al-Khudhair, Syed Abdul-Moiz Hasan, Nancy Salim, Juan Carlos Jaume

**Affiliations:** Department of Medicine, Division of Endocrinology, Diabetes and Metabolism and Center for Diabetes and Endocrine Research (CeDER), University of Toledo, College of Medicine and Life Sciences, 3000 Arlington Ave., M.S. 1186, Toledo, OH 43614 USA

**Keywords:** Hashimoto thyroiditis, Graves disease, Thyroid cancer, Tumor immunity, Macrophage-NK cells cross talk

## Abstract

**Background:**

Thyroid cancer and thyroid autoimmunity are considered opposite extremes of immune-responses. However, several studies have suggested that thyroid cancer coexists with autoimmune thyroid diseases like Hashimoto Thyroiditis (HT) and Graves disease (GD). We have shown that the risk of developing thyroid cancer is higher in patients with a silent form of autoimmune thyroid disease -Euthyroid Hashimoto Thyroiditis-(EHT).

**Methods:**

We analyzed data from 2633 consecutive patients with GD, HT, EHT and non-Autoimmune Thyroid Disease (Non-AITD) for the presence of Differentiated Thyroid Cancer (DTC). We further investigated the microenvironment, and cellular mechanism of protection from DTC in GD/EHT by ex-vivo aspirating infiltrates from thyroid samples. We also re-constituted in vitro the in-vivo microenvironment to mimic an in-vivo context. We isolated NK cells and differentiated macrophages into M1 and M2 phenotype from healthy human peripheral blood monocytes.

**Results:**

DTC was less frequent/aggressive in GD as compared to EHT or Non-AITD. Intra-thyroidal immune-cell profiling revealed differential Natural Killer (NK) cell activity and macrophage polarization in the settings of GD versus EHT. In GD, NK-cells were activated, and macrophages showed M1-like phenotype whereas, in EHT, NK-cells were less active and macrophages displayed M2-like phenotype. Furthermore, in vitro co-cultures of NK-cells with differentiated macrophage subsets revealed that the presence of activated NK (NA) cells favors M1 macrophages, boosts macrophage action and amplifies the innate defense mechanisms. Moreover, co-culture of M2 macrophages with NA, increases the cytotoxicity of NK-cells and favors a pro-inflammatory microenvironment that reverts the anti-inflammatory M2 towards pro-inflammatory M1.

**Conclusion:**

Surveillance innate immune-cells like Natural Killer (NK) cells and macrophages are complementary to each other in their actions. We discovered here that activated NK-cells in the background of the thyroid autoimmune disease, GD, drive macrophage differentiation to the M1/killer phenotype which in turn is cytotoxic to cancer cells and down regulates the M2/repair phenotype. Understanding the molecular basis of macrophage-NK cell interface in Thyroid Cancer, ETH and GD will open new vistas for immunopathology and therapeutic intervention. Macrophages/innate immunity can be modulated from M2 to M1 phenotype to help treat thyroid cancer as naturally done by GD.

**Electronic supplementary material:**

The online version of this article (10.1186/s40425-018-0483-y) contains supplementary material, which is available to authorized users.

## Introduction

Antitumor immune responses are often detected in human cancers, but in many cases, do not control tumor progression and/or may favor tumor growth [1, 2]. In organ-specific autoimmune diseases, immune responses are lethal to the target organ. Memory immune cells persist for life and facilitate chronic target destruction even under immunosuppressive conditions [3].

Thyroid cancer and thyroid autoimmunity seem to be situated at opposite extremes of the immune response spectrum. In thyroid cancer the immune response seems tolerant, allowing for tumor growth. In thyroid autoimmunity the immune response is destructive, usually leading to thyroid failure.

While known to immunologists for decades, it has surfaced in recent years that more effective immunotherapy of cancer is associated with autoimmunity [4, 5]. The interplay between autoimmunity and cancer is now taking center stage after the introduction of “immune check-point inhibitors” for cancer treatment. Favorable outcomes with these cancer immunotherapeutic drugs are now clearly associated with endocrine immune-related adverse effects like autoimmune thyroid disease and type 1 diabetes [6, 7].

In cancer development, progressive accumulation of genetic abnormalities renders cells malignant. The immune system seems to be allowing or even promoting cancer progression for some tumors [8]. While immune regulation in cancer seems to uphold development and progression, immune dysregulation in autoimmunity leads to tissue destruction and target elimination.

Thyroid cancer is usually surrounded by a significant number of immune “reactive” cells. Tumor associated leucocytes and macrophages (TAL and TAM) are frequently described in pathology reports of patients operated for thyroid cancer [9]. Macrophages play an important role in the progression/regression of cancer. Macrophage phenotype which stimulate tumor growth is M2/repair type whereas macrophages which inhibit/slow the tumor growth are M1/kill-type.

The nature of this leucocytic reaction is not well understood. Evidently, the fact that cancer can survive in this adverse immune microenvironment speaks for immune regulation.

We have recently shown that the risk of developing thyroid cancer is higher in patients with a silent form of autoimmune thyroid disease - Euthyroid Hashimoto Thyroiditis -. The risk is especially pronounced in patients with functional thyroids and undetectable/low titers of thyroid peroxidase antibodies (TPO), while diminished in patients with full thyroid failure and high TPO antibody titers [10, 11].

Some studies have suggested that thyroid cancer coexisting with another form of autoimmune thyroid disease - Graves disease - might be more frequent and aggressive as compared to that found in patients without Graves [12–14]. Our clinical observations argue against this hypothesis.

In the present study we investigated the roles of humoral and cellular autoimmunity in the development of thyroid cancer and the likelihood of less aggressive behavior in the setting of Graves disease, combining epidemiological and laboratory data.

## Material and methods

### Thyroid subjects

**Fig. 1:**
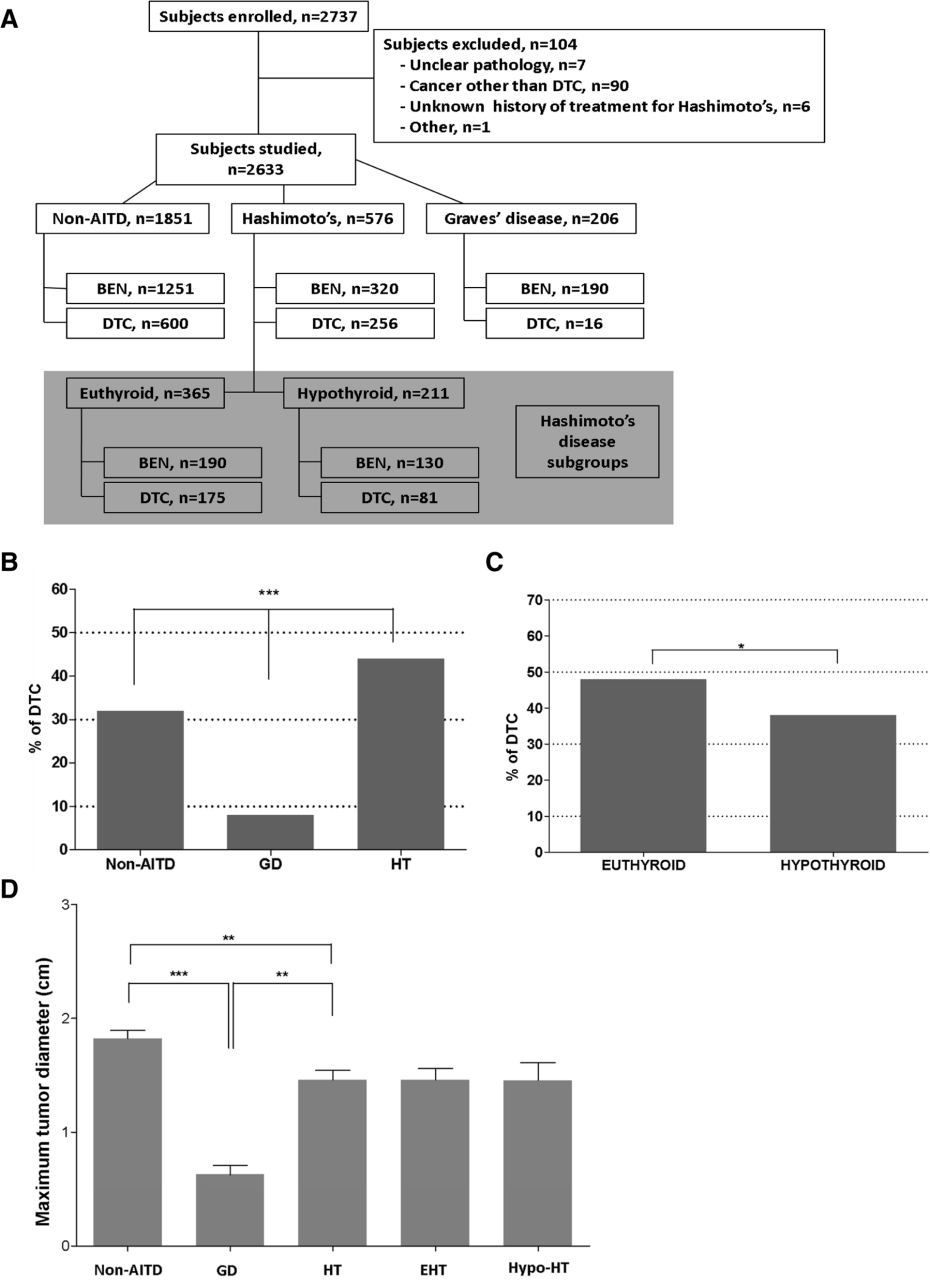
Relationship of Graves Disease (GD), Hashimoto Thyroiditis (HT) and Differentiated Thyroid Cancer (DTC) in patients undergoing thyroidectomy. Study design with benign (BEN) vs. malignant (DTC) outcome as related with either autoimmune (GD and HT with subgroups euthyroid and hypothyroid) or non-autoimmune (Non-AITD) (**a**). Bar graph for frequency of DTC in each group (**b**) and Hashimoto’s subgroups (**c**). Bar graph for distribution of DTC based on tumor size (**d**)

Our Thyroid Multidisciplinary Clinic is a large referral site for thyroid diseases. Patients referred for thyroid surgery include those with cytology positive or suspicious for malignancy on fine needle aspiration (FNA), and those with nodular goiter associated with compressive symptoms (such as dysphagia, shortness of breath or hoarseness). Random patients undergoing thyroid surgery had their thyroids ex-vivo aspirated in the operating room (Fig. 1a). A tissue sample from each of those patients was also snapped frozen in liquid nitrogen and stored for further analysis. Post-operative histology confirmed the presence of multinodular goiter, GD or HT, with or without DTC. We split our subjects with Hashimoto’s by histology into two subgroups: those with established hypothyroidism (abnormally high TSH and low free T4 preoperatively, treated with levothyroxine) and those with normal thyroid function (euthyroid Hashimoto thyroiditis) (Fig. 1a). We excluded patients with preoperative hypothyroidism which was a result of previous thyroid surgery or radioactive iodine treatment. In patients with Graves’ histology, we confirmed the diagnosis prior to surgery based on the presence of clinical hyperthyroidism with abnormally low TSH and high free T4, along with either exophthalmos, homogeneously enhanced Tc-99^m^ uptake or elevated titers of TSH receptor antibodies. Post operatively, established pathological characteristics for the diagnosis of multinodular goiter, thyroid cancer, Graves’ and Hashimoto’s were followed by our academic pathologists. The collection of patients’ tissue/data and subsequent analysis was approved by the University Human Subjects Institutional Review Board. Patients were invited to participate and signed consent pre-operatively to be part of this study.

For all patients, the following data was collected: gender, age and TSH, thyroid autoantibodies titers [TPO-Abs, Thyroglobulin antibodies (Tg-Abs), TSH receptor antibodies (TR-Abs), TSH receptor stimulating immunoglobulins (TSI)] when available, and surgical pathology report. TSH concentrations and thyroid autoantibodies titers were measured by chemiluminescent immunoassays.

We excluded patients on levothyroxine (LT4)-suppressive therapy (when used to prevent growth of a goiter or thyroid nodules), patients with prior exposure to radioactive iodine, prior thyroid surgery, or patients with incomplete records. No GD patients were treated with SSKI prior to surgery. Some patients received anti-thyroidal drugs prior to surgery but they demonstrated no statistical differences on the leukocyte infiltrates (see below).

### Intra-thyroidal leukocyte profiling

The resected thyroid glands were aspirated ex-vivo to collect infiltrating leukocytes. The red blood cells (RBC) in the aspirates were lysed by a brief hypotonic shock and the rest of the cells were suspended in RPMI-1640 media containing 10% Fetal Calf Serum (FCS) at 4^0^ C. The cells were stained for surface staining with fluorochrome-conjugated antibodies against human (Additional file 1: Table S2) or isotype controls in serum-containing media as described earlier [15].

Briefly, the cells were incubated with antibodies for 20 min on ice for surface staining, and then washed and fixed in 1% paraformaldehyde. Aliquots of cells were permeabilized with cytofix/cytoperm solution (BD Biosciences) and intracellular staining was performed using fluorochrome-conjugated antibodies against human (Additional file 1: Table S2). Cells were also stained with Hoechst 33342 (10 μg/ml for 2 h, Hoechst fluorescence, 350 nm excitation/ 450 nm emission, linear scale) used to gate live cells containing 2n-4n cellular DNA.

### In vitro stimulation/induction

Aliquots of thyroid infiltrating leukocytes/thyroid cells were stimulated (induced) in vitro with lipopolysaccharide (LPS) (100 ng/ml) and incubated in RPMI-1640 media with 10% FCS for 54 h at 37 °C in a 5% CO2 atmosphere. In last 6 h of induction, cells were treated with Golgi plug brefeldin A (1 μg/ml), (Sigma–Aldrich, St. Louis, MO) for intracellular cytokines analysis. The un-stimulated cells served as controls.

### NK/macrophage co-cultures

NK cells were isolated from healthy human peripheral blood using human NK isolation kit (Miltenyi Biotech, USA) as described by the manufacturers. Prior to co-culture, NK cells were plated in RPMI-1640 media plus 10% FCS with or without human IL-2 (50 ng/ml, Life Technologies Corporation, Grand Island, NY) at 37 °C for 16 h.

Macrophage subsets were differentiated from healthy human monocytes using M1 and M2 Macrophage Generation Medium (PromoCell, United States) as described by the manufacturer. M0 subsets represent macrophages differentiated from monocytes without adding M1/M2 polarizing cytokines to the media. Prior to co-culture, the macrophage subsets were washed three times with 10% FCS containing medium.

Resting (N0) or IL-2 activated NK (NA) cells were co-cultured with autologous macrophage subsets (M0, M1, and M2) at 1:1 ratio in RPMI-1640 media with 10% FCS for 72 h. Macrophages were analyzed for the expression of M1 or M2 lineage specific markers by flow-cytometry. NK cells were analyzed for NK cell marker (CD56) and intracellular cytokine expression (Granulysin, Granzyme B, Perforin, and IFNg) also by flow cytometry.

### Cytotoxicity assay

Natural Killer (N0 or NA) cells were co-cultured with K562 cells (ATCC, cat# CCL-243) or macrophage subsets to analyze the cytotoxicity of NK cells against K562 tumor target cells or macrophages. NK cells intracellular cytokine expression (Granulysin, Granzyme B, Perforin, and IFNg) was measured by flow cytometry. In another set of experiments, tumor target K562 cells or macrophage subsets were suspended in complete media (RPMI-1640+ 10%FCS) and stained with carboxyfluorescein diacetate succinimidyl ester (CFSE), followed by washing. Effector NK cells and target cells were plated together in 96-well plates in an effector-to-target (E:T) ratio of 10:1, 5:1, and 1:1. Effector cell populations were incubated with target cells for 4 h at 37 °C followed by staining with 7-AAD (BD Biosciences, San Jose, CA) at 37 °C for 30 min. Following incubation, cells were resuspended and CFSE+/7-AAD+ cells were counted by flow cytometry. A minimum of 25,000 target cells were acquired in 100 μl, which generally yielded 8,000–10,000 CFSE+ events. Background death of target cells alone was determined for each time point and subtracted from all data (Additional file 2 Figure S2 legend for more details).

### Flow cytometry

All samples were prepared in triplicates and the mean of the three samples was considered as an individual data. In each replicate, at least 25,000 live leukocytes were acquired. Samples were acquired in either BD LSRII/ FACSAria IIu or FACS Calibur flow cytometers (BD Biosciences, San Jose, CA). Analysis of fluorescence-activated cell sorting (FACS) data was done with FlowJo v. 10.3 software (Tree Star). Analysis of cell population was performed based on 4 step criteria (Additional file 3: Figure S1 legend for more details).

### Statistical analysis

Statistical analysis was done using the SAS MIXED procedure (version 9.3, SAS Institute, Inc., Cary NC, USA). Data was tested for normality by Kolmogorov-Smirnov test and transformed to natural logarithms or ranks as appropriate when not normally distributed. Comparisons between the two groups were done by Student’s t-test or Mann Whitney U test. Categorical data was analyzed with Fisher’s exact test and odds ratios (OR) with 95% confidence intervals (95%-CI) was calculated. The significant difference threshold was set at *p* ≤ 0.05 and *p* > 0.05 to *p* ≤ 0.10 indicated that a significance was approached. Data is presented as the mean ± SEM.

## Results

### Differentiated thyroid Cancer (DTC) risk in Graves disease

Differentiated thyroid cancer associated with Graves disease is believed to have more aggressive features [12–14]. As previously stated, our clinical experience argues against this hypothesis. Here we investigated the characteristics of DTC associated with GD, by reviewing our prospectively collected database of patients undergoing thyroidectomy, mostly for thyroid tumors (Fig. 1). We reviewed data for tumor size, tumor focality, extra-thyroidal extension, lymphatic or distant metastases, and need for reoperation. We also reviewed data for thyroid pathology and antibodies titers against Tg-Abs, TPO-Abs, TR-Abs also known as TBII and TSI-Abs.

As previously done for Hashimoto’s [10, 11, 15], we explored the relationship of DTC with all Autoimmune Thyroid Diseases (AITD). We analyzed the data from 2633 consecutive patients, recruited over 19 years (Fig. 1a). On histology, 206 patients were found to have GD, 576 had HT, while the remaining (*n* = 1851) were found not to harbor any autoimmune thyroid disease (Non-AITD). Subjects with HT were further subdivided into two subgroups: 211 subjects with hypothyroidism (Hypo-HT) and 365 subjects with normal thyroid function (EHT; all in Fig. 1a). Populations with either GD or HT were compared among themselves for the presence of DTC and with the Non-AITD population. The odds ratios (OR) and 95% confidence intervals (95% CI) for having DTC were calculated (Table 1). A similar comparison was made between subgroups of HT subjects with EHT and Hypo-HT and the results are presented in Table 1. We observed that DTC was less common in GD (Table 1 and Fig. 1b) and that within the HT group, EHT had a higher proportion of DTC (Table 1 and Fig. 1c).

**Table 1:** Differentiated thyroid cancer incidence and pathological features in patients with and without autoimmune thyroid diseases

	Non-AITD	HT	GD	EHT	Hypo-HT
DTC, n/total (%)	600/1851 (32.4%)	256/576 (44.4%)	16/206 (7.8%)	175/365 (47.9%)	81/211 (38.4%)
OR vs. Non-AITD 95% CI *P* value	n/a	**1.67** 1.38–2.02 < 0.0001	**0.18** 0.10–0.30 < 0.0001	**1.92** 1.53–2.41 < 0.0001	**1.30** 0.97–1.74 0.09
Comparisons Between subgroups	n/a	OR = **0.11** 95% CI = 0.06–0.18 *p* < 0.0001		OR = **1.48** 95%CI = 1.05–2.09 *p* = 0.029	
Size (cm) SEM	1.8 (1.7)	1.5 (1.3)	0.7 (0.3)	1.5 (1.2)	1.5 (1.4)
*p* value compared to Non-AITD	n/a	**0.008**	**< 0.001**	**0.029**	0.062
*p* value	n/a	**0.005**		0.831	
Macro n (%)	369 (66.4%)	142 (55.9%)	2 (12.5%)	98 (56.9%)	44 (57.8%)
OR vs. Non-AITD 95% CI *p* value	n/a	**1.56** 1.15–2.11 0.005	**13.8** 3.11–61.4 < 0.001	**1.49** 1.05–2.11 0.028	1.44 0.84–2.34 0.158
OR 95% CI *p* value	n/a	**8.88** 1.98–39.9 0.001		0.96 0.56–1.66 1.000	
FTC n (%)	46 (7.7%)	12 (4.7%)	0 0%	9 (5.1%)	3 (3.7%)
OR vs. Non-AITD 95% CI p value	n/a	1.69 0.88–3.24 0.112	2.77 0.16–46.9 0.623	1.53 0.73–3.20 0.316	2.16 0.66–7.11 0.254
OR 95% CI *p* value	n/a	1.69 0.10–29.8 1.000		1.41 0.37–5.35 0.758	
FVPTC n (%)	130 (21.7%)	56 (21.9%)	2 (12.5%)	36 (20.6%)	20 (24.7%)
OR vs. Non-AITD 95% CI *p* value	n/a	0.99 0.69–1.41 1.000	1.94 0.43–8.63 0.542	1.07 0.71–1.62 0.834	0.84 0.49–1.45 0.568
OR 95% CI *p* value	n/a	1.96 0.43–8.88 0.535		0.79 0.42–1.48 0.516	
Other PTC variants n (%)	12 (2.0%)	10 (3.9%)	0 0%	4 (2.3%)	6 (7.4%)

Abbreviations: *Non-AITD* subjects without any form of autoimmune disease by pathology, *HT* subjects with Hashimoto’s thyroiditis, *GD* subjects with Graves’ disease, *EHT* subjects with Hashimoto’s thyroiditis by pathology and normal thyroid function, *Hypo-HT* subjects with hypothyroidism due to Hashimoto’s disease, *Macro* differentiated thyroid cancer larger than 1cm in maximum diameter, *FVPTC* follicular variant of papillary thyroid cancer, *Other variants* other forms of papillary thyroid cancer, i.e. oncocytic, solid, tall cell variants, *Mets* distant metastases, *I-131* post-operative treatment with at least one dose of I-131

Comparison of thyroid cancer features with Fischer's exact test, between thyroid cancers found in the background of thyroid autoimmunity and those found in the absence of autoimmune thyroid diseases (Non-AITD). Odds ratios are reported as a comparison of the proportions between subjects with Non-AITD and subjects with autoimmune thyroid disorders

Odds ratios for the presence of DTC are estimated between subgroups of subjects with AITD and subjects without (Non-AITD). The odds ratios for DTC are estimated between different subgroups as well

Bold indicates a statistically significant difference between ratios

We then compared the features of tumor aggressiveness of DTC among GD, HT and Non-AITD subjects, based on tumor histology (presence of follicular thyroid cancer as compared to papillary thyroid cancer, percentage of subjects with follicular variant of papillary thyroid cancer or aggressive variants of papillary thyroid cancer), tumor size, extra-thyroidal extension of the tumor, lymph node metastasis, distant metastasis, use of I-131 therapy, and need for reoperation. We observed that GD was associated with less aggressive forms of DTC as compared to HT or Non-AITD (Table 1 and Fig. 1d). HT patients also had milder forms, compared to Non-AITD as measured by several, but not all parameters (Table 1).

Lastly, we checked for the presence of disease specific antibodies. Thyroid stimulating immunoglobulin (TSI) titers were available for 102 subjects with GD: 8 of them with DTC and 94 of them with benign disease. The presence of elevated TSI titers was similar in both, DTC (5/8) and benign disease (60/94), OR 0.94 (95% CI 0.21–4.20, *p* > 0.99). Similarly, data on TR-Abs was available on 45 subjects with GD, 5 subjects with DTC and 40 subjects with benign disease. The presence of elevated titers of TR-Abs was similar in both groups with DTC (4/5) and benign disease (32/40), OR 1.00 (95% CI 0.10–10.2, *p* > 0.99).

Data was available on TPO antibodies titers in 136 patients with GD and in 295 patients with HT. For analysis, we split our subjects into two groups, those with high titers of TPO Abs (> = 100 IU/L) (TPO+) and those with low or absent titers (< 100 IU/L) (TPO-). We compared the frequency of DTC in GD and HT patients as a function of auto-antibody titers. Low/absent TPO titers were associated with a higher risk for DTC both in GD (OR 3.7, 95% CI 1.1–12.6, *p* = 0.043) and HT (OR 2.3, 95% CI 1.4–3.7, *p* < 0.001). This association persisted when we compared these proportions in Hypo-HT (OR 6.5, 95% CI 2.9–14.5, *p* < 0.001) but not in EHT (OR 1.3, 95% CI 0.7–2.5, *p* = 0.43) (Additional file 4: Table S1).

### Intra-thyroidal immune profiling revealed differential NK cell activity and macrophage polarization

**Fig. 2:**
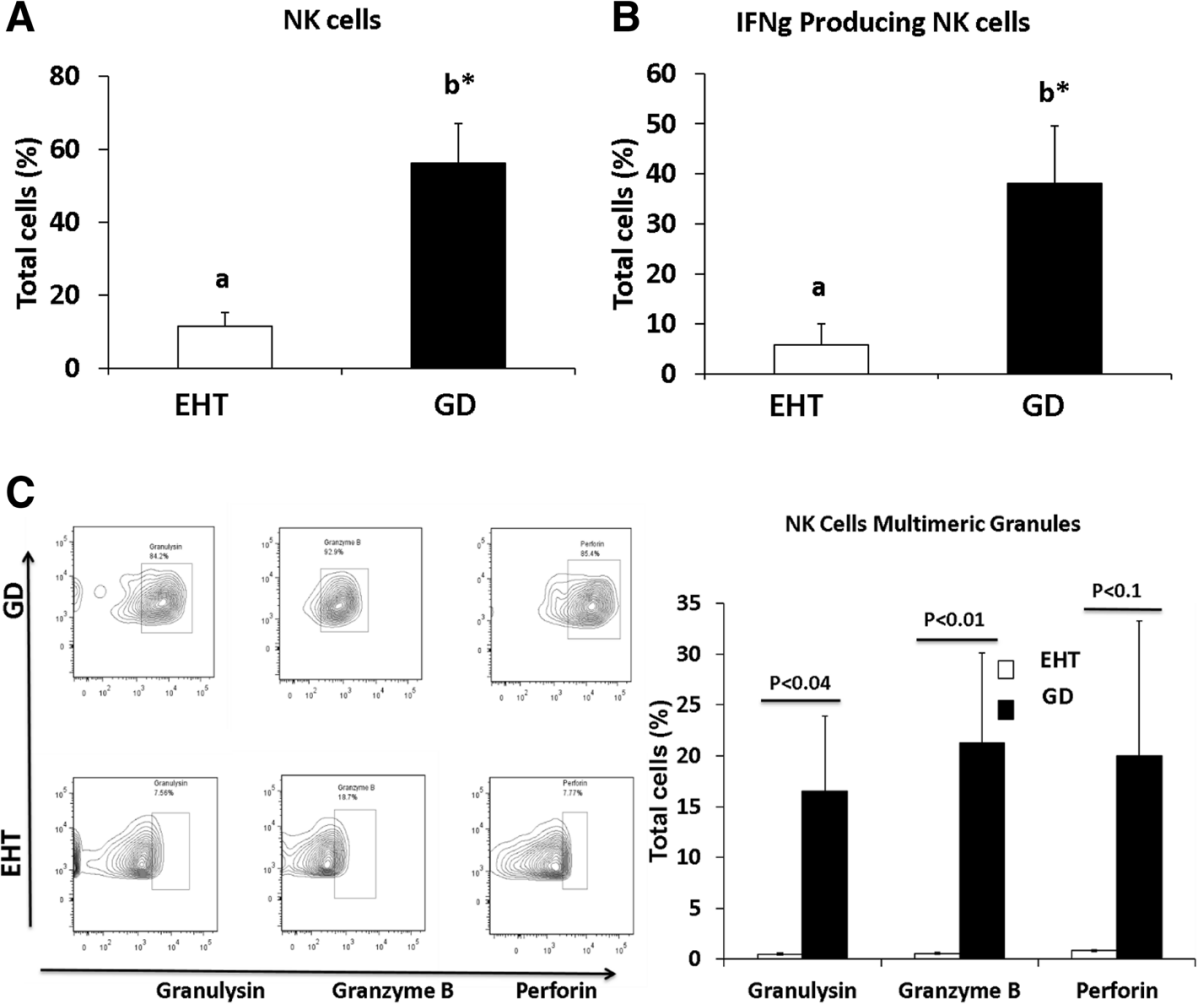
Flow cytometry analysis of natural killer (NK) cells. Bar graphs of statistical analysis of NK cell present in patient samples (**a**) and functionality as measured by Interferon gamma (INFg) from patients with Euthyroid Hashimoto Thyroiditis (EHT) (*n* = 8) and Graves disease (GD) (*n* = 8) (**b**). Flow cytometry raw data sample of NK cells in EHT or GD and bar graph statistical quantification based on production of cytotoxic enzymes: Granulysin, Granzyme B and Perforin (**c**). Statistical significance was determined by using t-test: two samples assuming unequal variance. ^**a b**^ depicts the difference (*P* < 0.05) between the groups

We further investigated the microenvironment and cellular mechanism of protection from DTC in GD. Since failure of tumor immunity allows for cancer growth, we reasoned that immune profiling of the thyroid gland immune infiltrates might unravel the cause for this failure. We compared the intra-thyroidal mononuclear cell infiltrates in patients with EHT (*n* = 8) and GD (*n* = 8). Our data revealed that NK cells identified as CD3-CD56+ cells were significantly more abundant in GD as compared to EHT (Fig. 2a). We then compared the activation status and cytotoxic profile of these NK cells in both conditions. The NK cells carrying high levels of interferon gamma (IFNg), which reflects their activation status, were significantly higher in numbers in GD as compared to EHT (Fig. 2b). The activated NK cells also produced significantly higher amounts of cytotoxic granules in GD than in EHT including Granulysin, Granzyme B and Perforin respectively (Fig. 2c).

**Fig. 3:**
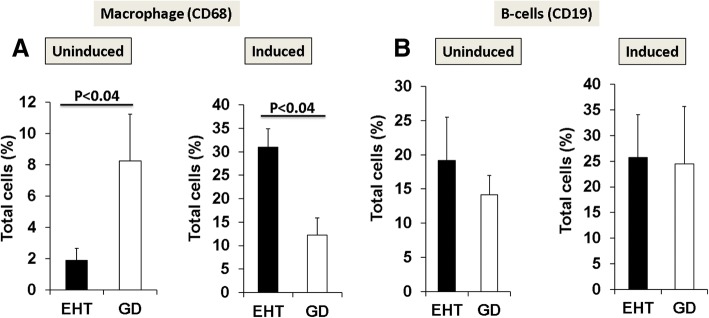
Macrophage and B cell comparison under induction/stimulation. Macrophages in EHT (n = 8) and GD (n = 8) before and after induction/stimulation (**a**) as analyzed by flow cytometry were compared with B cells from same patients (**b**) induction/stimulation under high dose of LPS (100 ng/ml) for 54 h

**Fig. 4:**
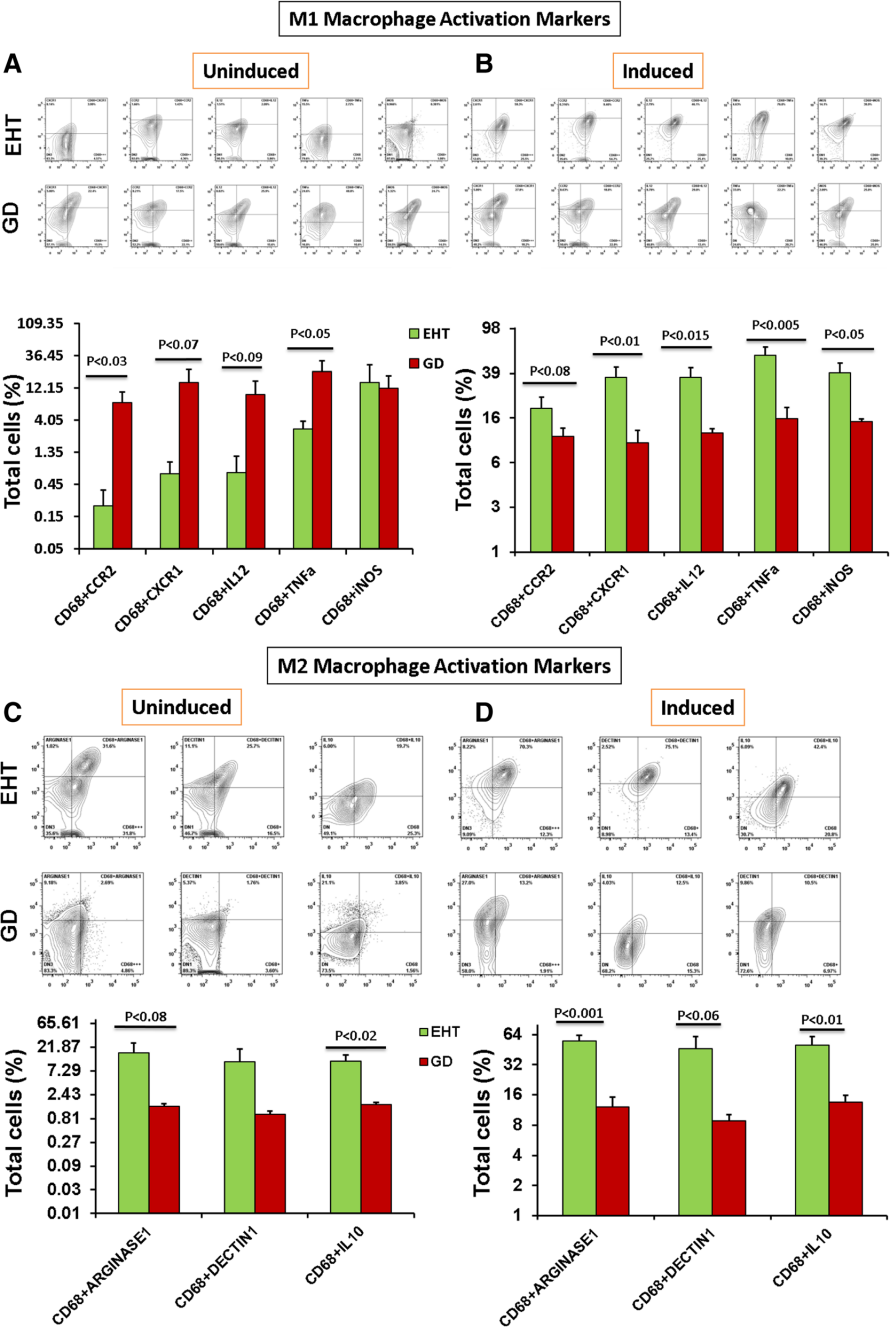
Intra-thyroidal immune profiling of M1 and M2 macrophage polarization using Flow Cytometry Analysis. Flow cytometry analysis of M1 macrophages (FACS) contour plots (Upper panel) of representative patients and Bar graphs (Lower panel) of statistical analysis of leukocyte specimens from patients with Euthyroid Hashimoto Thyroiditis (EHT) (*n* = 8) and Graves disease (GD) (*n* = 8). Leukocyte specimens gated for CD3-ve and subsequently sorted for macrophages (CD14 and CD68). Macrophages were re-gated for the M1 macrophage activation marker Viz. CCR2, CXCR1, IL12, TNFa and iNOS, Uninduced (**a**) and Induced (**b**) are shown. Macrophages were again re-gated for the M2 macrophage activation marker Viz. Arginase 1, Dectin 1 and IL10. Uninduced (**c**) and Induced (**d**) are shown. Statistical significance was determined by using t-test: two samples assuming unequal variance

Macrophages identified as CD68+ cells were also significantly more abundant in GD as compared to EHT (Fig. 3a). Concentration of B cells (CD19) was not different in either condition (Fig. 3b). Humoral responses (CD19) in EHT and GD were quantitively similar in both diseases (Fig. 3b). By sorting macrophages into M1 and M2 subpopulations, we found that M1 macrophages present in GD were secreting significantly higher levels of chemokines (CCR2 and CXCR1) as compared to M1 macrophages present in EHT (Fig. 4a). We also observed that cytokines that characterize the M1 pro-inflammatory phenotype, TNFa and IL-12, were higher in GD than in EHT, significantly for TNFa and with a trend towards statistical significance for IL-12 (Fig. 4a). In contrast, M2 macrophages were significantly more abundant in EHT than in GD as detected by expression of ARGINASE1 and DECTIN1 (Fig. 4c). Also, M2, anti-inflammatory cytokine, IL10 was significantly higher in EHT than in GD (Fig. 4c).

We next activated (induced) the intrathyroidal samples from GD and EHT patients in vitro with high dose of LPS (100 ng/ml) for 54 h. The LPS activation significantly increased the combined expression of M1 phenotype markers (CD68 + CCR2, CD68 + CXCR1, CD68 + IL12, CD68 + iNOS and CD68+ TNFa) and M2 phenotype markers (CD68 + ARGINASE1, CD68 + IL10, and CD68 + DECTIN1) in EHT; whereas there were non-significant effects on the activated M1 or M2 macrophage phenotypes in GD samples (Fig. 4b and d). In the setting of EHT, M1 and M2 macrophage phenotypes were not only different but also had a higher degree of plasticity as compared to GD setting (Fig. 4). We also observed that induction of intrathyroidal samples with higher doses of LPS, induced macrophage proliferation (not shown) which was significantly increased in EHT as compared to GD.

LPS, like IFNg, acts as a pro-inflammatory stimuli for M1 macrophages, and strongly promotes IL-12 mediated T helper 1 responses [16]; but they (M1s) also induce innate anti-tumoral responses through activation of resting NK cells, which eliminate tumor cells and maintain a pro-inflammatory microenvironment. Therefore, we investigated NK cells further.

### Functional outcomes are mediated by NK cell-macrophage cross talk

**Fig. 5:**
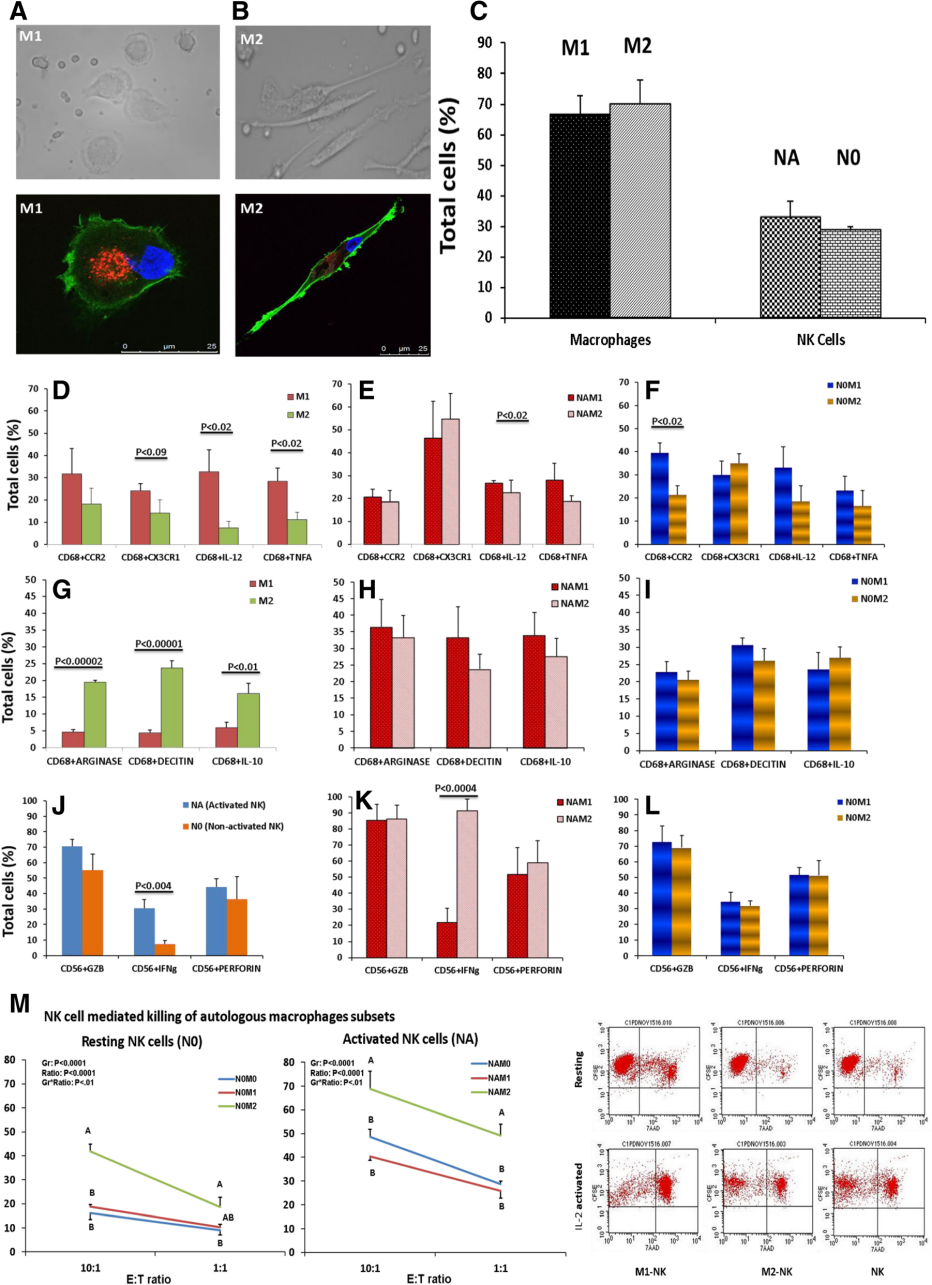
NK-Macrophage crosstalk. Peripheral Blood M1-M2 differentiation and quantification of final product. M1 (**a**) and M2 (**b**) structural phenotype shown. Macrophages were labeled with CD68-PE (Red), Cytoskeleton stained with phalloidin-Alexa Fluor 488 (Green) and nuclei stained with DAPI (Blue). Bar graph of proportions of M1 and M2 as well as NK cells in active (NA) or resting (N0) form as quantified by flow cytometry (**c**). Naïve/resting NK cells were activated by using IL-2 at the dose rate of 50 ng/ml. Macrophages were differentiated from human PBMCs into M1 and M2 macrophages. Differentiated macrophages (M1 and M2) and NK (NA and N0) cells were co-cultured. All the experiments were executed in triplicate and mean of the three were considered as an individual observation (*n* = 3–6). Autologous co-cultures of M1/M2 macrophage with NA/N0 NK cells were stained with M1/M2 phenotype markers. Phenotypic characterization of differentiated macrophages (M1 and M2) were done using Flow cytometer. Single live cells were gated and subsequently sorted for macrophages (CD68) and re-gated for the M1 macrophages activation marker Viz. CCR2, CX3CR1 for surface chemokine and IL-12 and TNFa for intracellular cytokines (**d**-**f**). In the same autologous co-culture experiment the macrophages were re-gated for CD68 and gated for M2 macrophage activation marker Viz. Arginase 1, Dectin 1 and IL-10 (**g**-**i**). Again in the same autologous co-culture experiment single cells were gated for NK cell markers (CD56+ CD3-) and subsequently sorted for intracellular cytokines and multimeric complexes viz. GZB, IFNg, and Perforin (**j**-**l**). Statistical significance was determined by using t-test: two samples assuming equal variance. NK cells were co-culture against macrophages at different Effector to Target (E:T) ratios: Resting and activated NK (N0 and NA) cells cytotoxicity against macrophage (M0, M1 and M2) is shown (**m**). Cytotoxicity was assessed in flow cytometer using CFSE-FITC for alive and 7 Aminoactinomycin D (AAD)-PE for dead cells. ^**AB**^ depict the difference between the groups within a ratio and ^**ab**^ depict the difference (*P* < 0.05) among the ratios within a group

To dissect the cross talk between NK cells and macrophages in the setting of GD and EHT, we re-constituted in vitro the in-vivo microenvironment. We co-cultured macrophages with activated NK (NA) and resting NK (N0) cells to mimic, as close as possible, the in-vivo context. We isolated human peripheral blood monocytes and differentiated them into macrophage subsets of M1 or M2 phenotypes (M1 and M2 structural phenotypes shown in Fig. 5a-b), and then co-cultured them with autologous NK cells. The proportion of M1 and M2, as well as NK cells in active (NA) or resting (N0) form that we were able to differentiate is shown in Fig. 5c as quantified by flow cytometry.

To prove our hypothesis of whether NK cells derived IFNg is sufficient to polarize macrophages from M2 to M1 phenotype, we generated an autologous co-culture of M1-NA, M2-NA, M1-N0 and M2-N0. Autologous co-cultures of M2 macrophages with NA/N0 NK cells were stained with M1 phenotype markers. The expression profiles were recorded by using flow cytometry and revealed that autologous co-cultures of M2 with NA/N0 upregulate pro-inflammatory chemokines (CXCR2 and CX3CR1) and cytokines (IL12 and TNFa) in M2 macrophages which were comparable to M1 macrophage expression profiles (Fig. 5 d-f). Similar results were observed in autologous co-cultures of M1 macrophages with NA/N0 NK cells which were stained for M2 markers (Fig. 5 g-i). Interestingly, we also observed that M2 co-culture with activated NK (NA) cells significantly increases the IFNg expression by NK cells (Fig. 5 k).

### NK cell mediated killing of autologous macrophages subsets

To study the cytotoxic role of NK cells upon autologous macrophage interactions, we co-cultured the different macrophage subsets with NK cells at 1:10 and 1:1 ratio. We observed a reciprocal relationship between NK cell activation status and the macrophage’s phenotypic responses. We observed cytotoxicity towards all three subsets of macrophages (M0, M1 and M2), but cytolysis was significantly higher for M2 macrophages (Fig. 5 m). It has been reported that NKP46, an NK cell activating receptor plays a major role in the killing of M0 and M2 macrophages [17]. NK cells co-cultured with M1 macrophages express a higher amount of NKP46 as compared to NK co-cultured with M0 and M2 macrophages (not shown). In another experiment, when unpolarized M0 cells were co-cultured with activated NK cells either in presence of M1 or M2 differentiation media, we observed that activated NK cells promoted the differentiation of M0 to M1 phenotype only (data not shown). Thus, in the presence of M1differentiation media, soluble factors activated NK cells which in turn favored M1 dominance, as seen in the background of GD. However, in the absence of NK cells, soluble factors favors M0 differentiation towards M2 dominance, as in EHT.

### Cytotoxic activity of NK cells against K562 target cells

We then tested the cytotoxicity of NK cells (*Effector* cells) against K562 tumor cells (*Target* cells). We confirmed that activated NK cells (NA) significantly increased cytotoxic activity against K562 cells as compared to resting NK cells (N0) (Additional file 2: Figure S2).

## Discussion

Inflammation is associated with cancer in most organs. In the thyroid gland, inflammation has been linked to DTC by some, but not all scholars. We have previously shown that EHT, a silent state of chronic inflammation, is associated with the presence and severity of thyroid cancer [10, 15]. However, controversy remains regarding the potential association of thyroid cancer and its severity in GD [12–14]. Some groups suggest an association and even postulate an increased mortality in patients with DTC associated with GD. Our clinical observations argue against this hypothesis.

HT and EHT are phenotypically different thyroid autoimmune diseases. In HT, thyroid glands have lymphocytic infiltrates functionally different than those accompanying thyroid cancers in EHT. Lymphocytes present in HT are mainly effector cells while lymphocytes accompanying thyroid cancer in EHT appear to be inactive and under immune regulation [15]. HT is also functionally different than GD, however both are symptomatic autoimmune diseases of the thyroid.

As with HT, our results show that GD seems to be infrequently associated with thyroid cancer (as opposed to EHT). When thyroid cancer coexists with GD, a less aggressive form of differentiated thyroid cancer is more common. The immune microenvironment of cancer coexisting with GD appears to be more pro-tumor elimination, than in patients without GD. We recognize the possibility of selection bias as a patient with GD may be diagnosed earlier than patients with silent or Non-AITD. However, the different immune process present in GD and characterized here is consistent with the observed clinical outcome.

The presence of a strong humoral autoimmune response in the form of high TPO-Ab, Tg-Ab and TR-Ab titers appears to be protective from DTC in patients with GD as judged by our epidemiological observation. To the contrary, the absence of high autoantibody titers appears to confer some risk for DTC in the setting of GD. TSI however were equally present. TSI have the ability to engage the TSH receptor and induce hyperthyroidism, which is clinically associated with benign outcomes. However, the number of patients checked for TSI in this study does not allow for any statistical conclusion.

We also demonstrate here that cellular immune infiltrates accompanying thyroid cancer in the background of GD have higher proportions of NK cells and significantly higher numbers of M1 macrophages as compared with EHT. On the other hand, the immune infiltrates in EHT are low in NK cells and M1 macrophages, but have a higher M2 macrophage presence. In particular, we noticed that the NK cell population in GD differs from the one in EHT, in that NK cells in GD are mostly activated. Measurements of cytotoxic multimeric complexes of activated NK cells (Granulysin, Granzyme B and Perforin) as well as INFg were all high in the GD immune infiltrates. Despite the differences in the humoral responses between GD and EHT noted above, the quantification of B cells (CD19) was not different in either group.

As mentioned above, macrophages were more abundant in lymphocytic infiltrates of GD than of EHT patients. Although disproportionally, induction of the intra-thyroidal immune infiltrates with lipopolysaccharide (LPS) increased the number of macrophages in both GD and EHT. It is well known that the stimulation of resting macrophages (M0) with Th1 cytokines (i.e., IFNg) or TLR4 ligands (i.e., LPS) induces the classical polarization towards M1, which displays strong microbicidal and tumoricidal properties and preferentially promotes inflammatory responses. In contrast, the alternative polarization towards M2 macrophages is induced by the Th2 cytokine IL-4 [18].

Specifically, M1 macrophages were significantly more abundant in the background of GD while M2 were significantly more abundant in the background of EHT. Both M1 and M2 phenotypic markers increased post-induction in EHT, but induction had a minimal effect on the macrophages in GD; indicative of higher degree of plasticity of macrophages in the background of EHT.

It has been reported that M1 (but not M0 or M2) macrophages can activate NK cells. Soluble mediators as well as cell-to-cell interactions of M1 with NK cells activate the later into its cytotoxic state [19]. Co-culturing NK activated (NA) or resting (N0) cells with M2 macrophages demonstrated distinct phenotypic outcomes. Co-culturing NA/N0 cells with M2 macrophages increased the expression of M1 phenotype surface chemokines (CCR2, CX3CR1), and intracellular cytokines (IL-12, TNFa). We however observed a novel phenomenon. Co-culturing activated NK cells (NA) with M2 macrophages significantly upregulated the IFNg expression of NK cells (Fig. 5 k). The change in the expression profile from M2 to M1 phenotype and the expression of IFNg by NK cells was inter-related, which may be explained as NA and M2 co-cultures upregulate CD80, sustaining Th1 responses, and CD48, a major ligand for 2B4, both expressed by M2 macrophages during co-culture [19].

2B4, an NK cell activating receptor binds with CD48 on macrophages and triggers NK cell cytotoxicity by enhancing degranulation and release of IFNg, which in turn sustains CD48 expression by M2 macrophages [19]. The more aggressive response of NK cells, when co-cultured with M2, apparently resulted from significant NK-cell expressed IFNg, which upregulated pro-inflammatory cytokines (IL-15/IL-15Rα) complex, and IL-1β by M2 macrophages [19]. It is quite interesting that the M1 macrophages were tolerant towards second IFNg stimulation in terms of IL-1β transcription, whereas M2 macrophages were strongly upregulated in first stimulation and equally responsive to second IFNg stimulation which potentially transcribes higher levels of IL-1β than in M1 [19]. Although IL-1β has pro-inflammatory effects, it also actively participates in NK cell activation as IL-1β upregulates NK cell activating receptor NKp44 [19]. Therefore, the IFNg dominant microenvironment favored the M2 macrophages polarization towards M1.

Although it needs further investigation, M2 macrophages seem to respond better to IFNg as compare to M1. We have observed the same events in GD, where highly activated NK cells created a pro-inflammatory M1 microenvironment as opposed to the EHT condition where M2 non-inflammatory dominance showed a significantly higher degree of macrophage plasticity after induction with LPS. With these experiments, we have categorically explained that in natural disease conditions, macrophage plasticity is microenvironment/NK dominance dependent. We can exploit the M2 macrophage’s plasticity by using TLR4 agonist (LPS in this study) which has the capacity to create a pro-inflammatory microenvironment and re-educate the M2 macrophages towards M1 phenotype.

The increase in cytotoxicity of NK cells exposed to M1-differentiation media may be because of M1 macrophages upregulating CD48, which is a major ligand for 2B4 (expressed by NK cells), so NK-M1 co-culture modulated by 2B4-CD48 interaction may increase the NK cells IFNg expression [19]. Therefore, the production of IFNg may act as a major inductor of NK cell cytotoxicity towards tumor target cells. Thus, in the setting of NK activation, the final differentiation outcome is M1 dominance, as seen in the background of GD. Moreover, the M2 dominance, as seen in EHT, seems to affect recruitment of NK cells at the cancer site.

Therefore, the tumor microenvironment seems to play a significant role in limiting the activation of NK cells that can kill tumor cells through IFNg [20]. M2 macrophages can be modulated to the M1 phenotype and such activation can cause tumor recession [20–22]. M1 macrophages activate NK cells and induce IFNg production which in turn plays a key role in inducing M1 polarization [18]. Others have reported on partial reversal of M2 markers when exposed to activated NK cells [19]. Remarkably, our data shows that M1 co-cultured with NK cells revert an established M2 phenotype to its M1 cytotoxic potential.

Although in this paper we focused on DTC, the most malignant form of thyroid cancer (anaplastic) develops within a matrix of macrophages. Anaplastic thyroid cancer is reported to distinctively grow in a very dense network of interconnected macrophages (flagellating like M2 s as in Fig. 5b) in direct contact with intermingled cancer cells [23]. Even anaplastic thyroid cancer metastasis seems to depend on local macrophages for establishment and progression [24].

But, more important than the M2 association with thyroid cancer is the discovery that activated NK cells can drive M1 differentiation, which in turn is cytotoxic to cancer cells and down-regulates M2 s. In fact, immunomodulatory strategies for activating NK cells are already in clinical trial. Flagellin (another TLR ligand) for example, induces activation of NK cells and has less adverse effects compared with LPS [25]. Therefore, immunomodulatory flagellin could potentially be an alternative treatment option for cancers dependent on M2 support (like anaplastic thyroid cancer).

This is the first time thyroid cancer behavior in the background of GD is shown to depend on the NK-M1 dynamics. Cancer seems capable of disrupting the M1 dominance by tipping the macrophage plastic balance towards the M2 phenotype. Fortunately, this process seems to be therapeutically reversible, in that TLR5 agonist (flagellin) may reverse the M2 phenotype (data not shown).

We concluded that in the setting of EHT, the macrophage phenotype was not just different but also had a higher degree of plasticity than in the GD setting. In GD, the presence of functionally active NK cells and higher M1/M2 macrophage ratio may provide Graves’ patients with a more effective form of tumor immunity. However, in the absence/low count of active NK cells, macrophage plasticity allows M0 to differentiate to the M2 phenotype, which may explain the higher risk of thyroid cancer in EHT.

Overall, studying the tumor microenvironment in thyroid cancer may help us understand the different clinical behaviors of thyroid cancers with similar histological and molecular signatures. Moreover, clinical behavior of other cancers may likely be related to these specific immune cell players. In the era of cancer immunotherapy, knowledge about tumor immunity is no longer basic but clinical.

## Additional files

Additional file 1: **Table S2.** A) Surface staining using fluorochrome-conjugated antibodies against human. B) Intracellular staining fluorochrome-conjugated antibodies against human. (PDF 35 kb)

Additional file 2: **Figure S2.** Cytotoxicity of NK cells against K562 cancer target cells. (PDF 404 kb)

Additional file 3: **Figure S1.** A) Gating strategies for flow cytometry analysis from representative patients with EHT and GD. B) In-vitro NK cells –Macrophages crosstalk gating strategies. (PDF 1122 kb)

Additional file 4: **Table S1.** Subjects with differentiated thyroid cancer split in subgroups based on thyroid peroxidase antibodies titers. (PDF 32 kb)
