# Supplementary material for: Nature of coexisting thyroid autoimmune disease determines success or failure of tumor immunity in thyroid cancer
Source: J Immunother Cancer. 2019 Jan 7;7:3. doi: 10.1186/s40425-018-0483-y (PMC6323721; doi:10.1186/s40425-018-0483-y)
Supplement: Supplementary file 1 — Table S2. A) Surface staining using fluorochrome-conjugated antibodies against human. B) Intracellular staining fluorochrome-conjugated antibodies against human. (PDF 35 kb) [file 40425_2018_483_MOESM1_ESM.pdf]

List of antibodies used for flowctometry:

A) Surface staining using fluorochrome-conjugated antibodies against human

CD45 (clone HI30, FITC), (BD Biosciences, San Jose, CA)

CD3 (clone HIT3a, APC), (BD Biosciences, San Jose, CA)

CD192/CCR2 (clone K036C2, PerCP/Cy5.5), (BioLegend San Diego, CA)

CX3CR1 (clone 2A9-1, APC), (BioLegend San Diego, CA)

CD14 (clone M5E2, PE), (BD Biosciences, San Jose, CA)

CD19 (clone HIB19, PE Cy5), (BD Biosciences, San Jose, CA)

CD56 (clone B159, PE Cy7), (BD Biosciences, San Jose, CA)

CD335 (NKp46) (Clone 9E2/NKp46, BV650), (BD Biosciences, San Jose, CA)

B) Intracellular staining fluorochrome-conjugated antibodies against human

CD68 (clone Y1/82A, PE), (BioLegend San Diego, CA)

TNFa (clone MAb11, APC/Cy7), (BioLegend San Diego, CA)

Interleukin (IL)-10 (clone JES3-9D7, PE/Cy7), (BioLegend San Diego, CA)

Interleukin (IL)-12/23p40 (clone C11.5, Alexa Fluor 488), (BioLegend San Diego, CA)

Perforin (clone B-D48, PerCP/Cy5.5), (BioLegend San Diego, CA)

Granzyme B (clone GB11, PE), (BD Biosciences, San Jose, CA)

Granulysin (clone RB1, Alexa Fluor 488), (BD Biosciences, San Jose, CA)

IFNg (clone 25723.11, FITC), (BD Biosciences, San Jose, CA)

Arginase 1/ARG1 (Polyclonal Sheep IgG), (R&D Systems, Minneapolis, MN)

Dectin-1/CLEC7A (IgG2B Clone 259931), (R&D Systems, Minneapolis, MN)

iNOS (rabbit polyclonal, FITC, Biorbyt LLC, San Francisco, CA).

Hoechst 33342 (Invitrogen, Carlsbad, CA)
