# Supplementary figures and images for: Nature of coexisting thyroid autoimmune disease determines success or failure of tumor immunity in thyroid cancer
Source: J Immunother Cancer. 2019 Jan 7;7:3. doi: 10.1186/s40425-018-0483-y (PMC6323721; doi:10.1186/s40425-018-0483-y)

# Cytotoxicity of NK cells against K562 cancer target cells

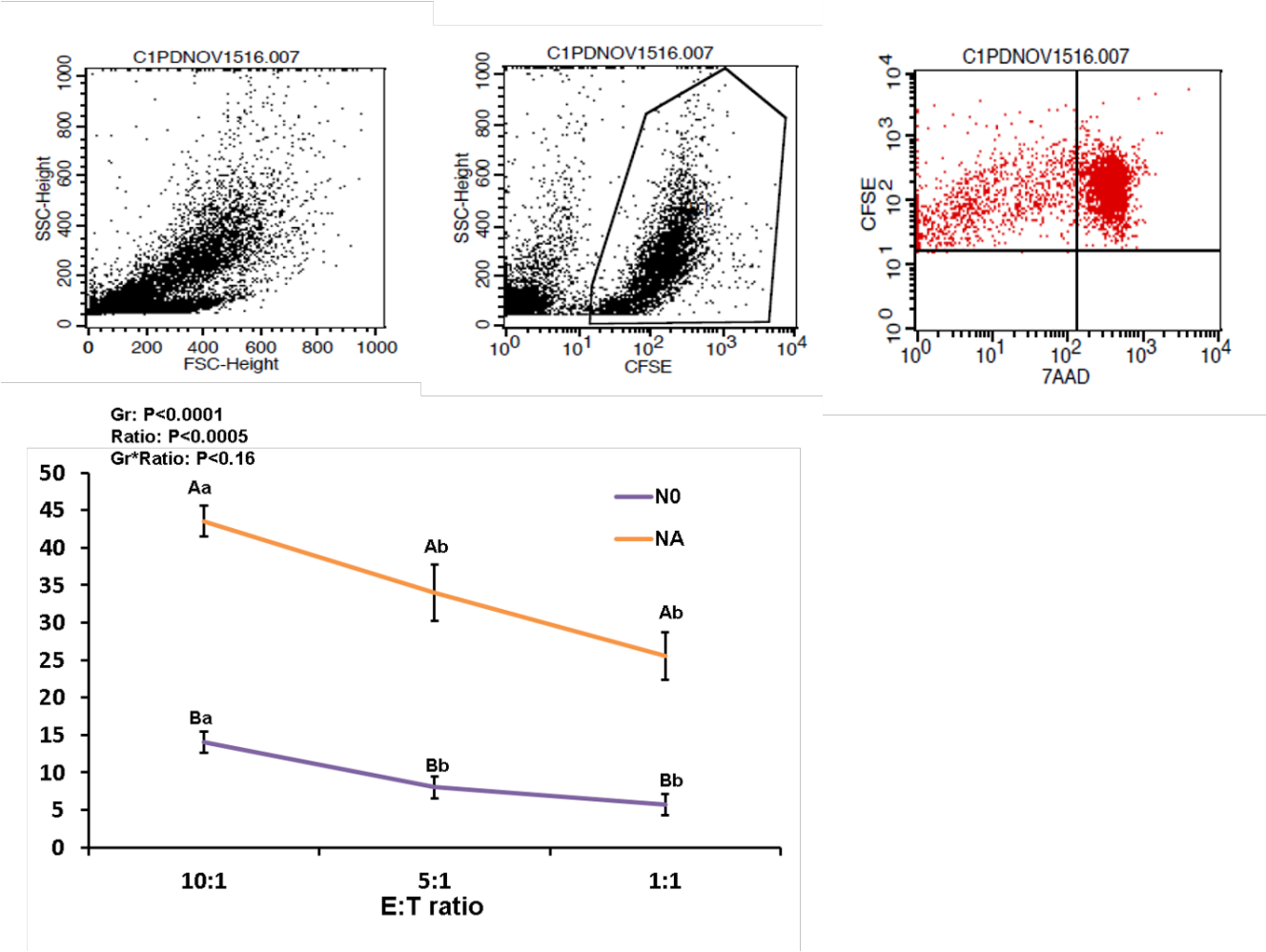

Supplement Fig. 2.

Supplement: Supplementary file 2 — Figure S2. Cytotoxicity of NK cells against K562 cancer target cells. (PDF 404 kb) [file 40425_2018_483_MOESM2_ESM.pdf]
