# Supplementary material for: Nature of coexisting thyroid autoimmune disease determines success or failure of tumor immunity in thyroid cancer
Source: J Immunother Cancer. 2019 Jan 7;7:3. doi: 10.1186/s40425-018-0483-y (PMC6323721; doi:10.1186/s40425-018-0483-y)
Supplement: Supplementary file 3 — Figure S1. A) Gating strategies for flow cytometry analysis from representative patients with EHT and GD. B) In-vitro NK cells –Macrophages crosstalk gating strategies. (PDF 1122 kb) [file 40425_2018_483_MOESM3_ESM.pdf]

A) Gating strategies for flow cytometry analysis from representative patients with EHT and GD

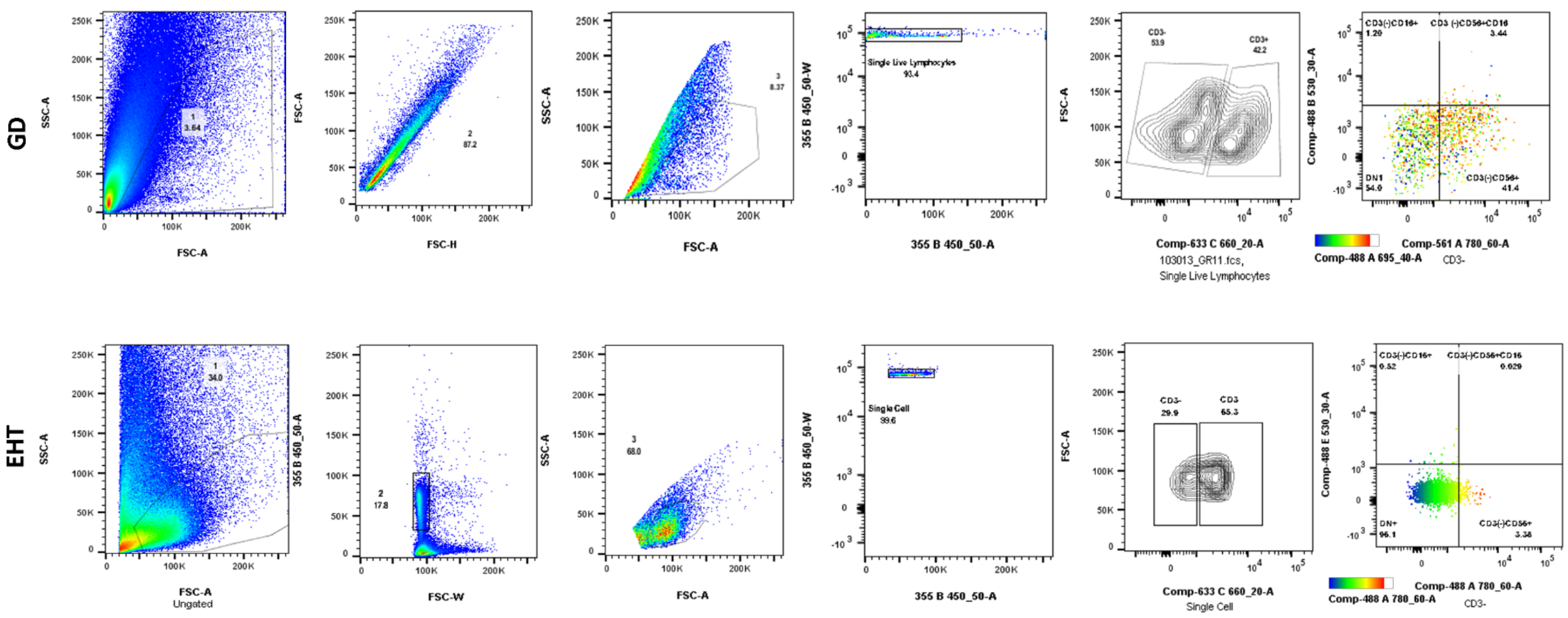

Supplement Fig. 1A.

B) In-vitro NK cells –Macrophages crosstalk gating strategies:

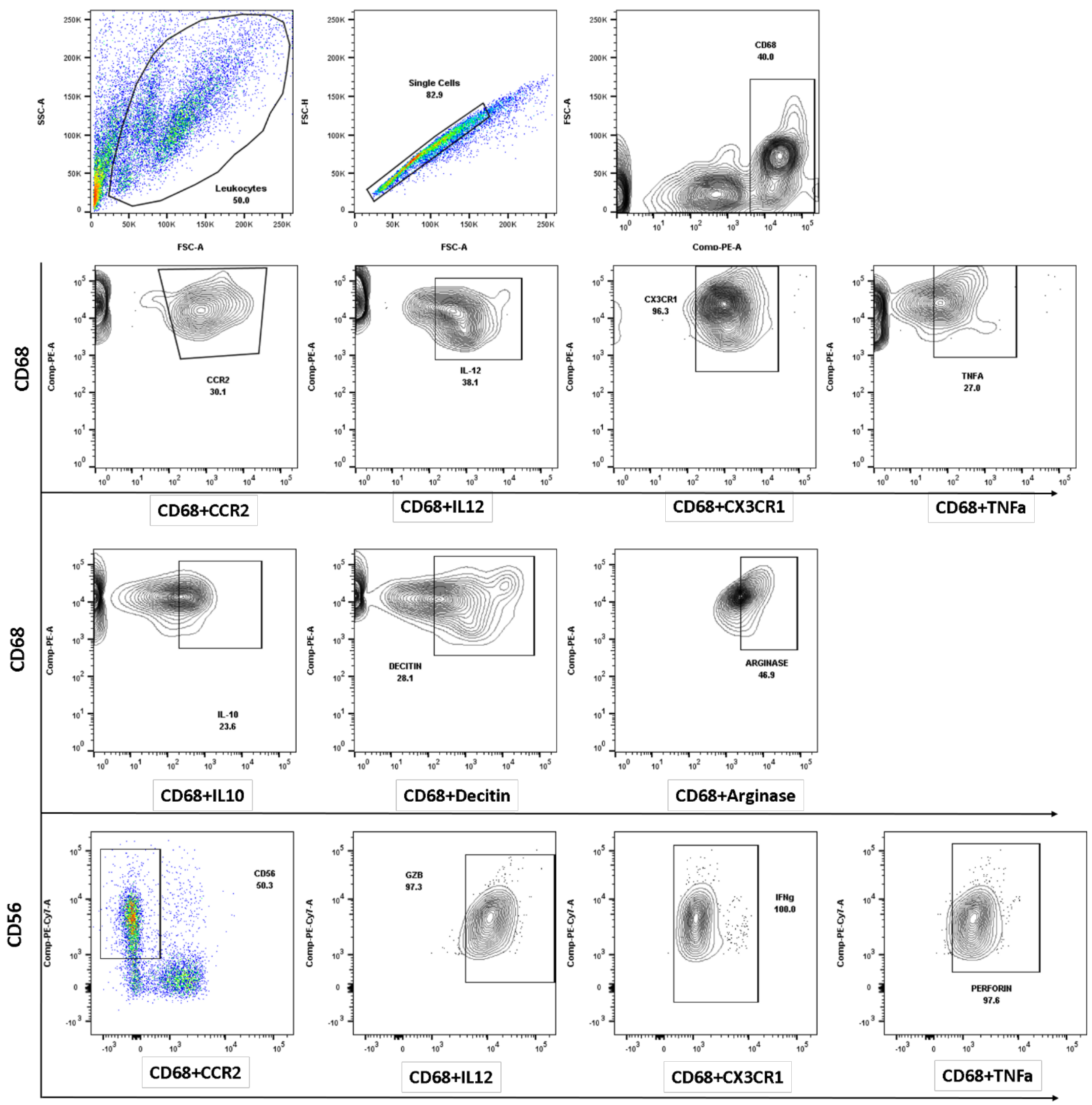

Supplement Fig. 1B.
