# Supplementary material for: Nature of coexisting thyroid autoimmune disease determines success or failure of tumor immunity in thyroid cancer
Source: J Immunother Cancer. 2019 Jan 7;7:3. doi: 10.1186/s40425-018-0483-y (PMC6323721; doi:10.1186/s40425-018-0483-y)
Supplement: Supplementary file 4 — Table S1. Subjects with differentiated thyroid cancer split in subgroups based on thyroid peroxidase antibodies titers. (PDF 32 kb) [file 40425_2018_483_MOESM4_ESM.pdf]

Supplemental table 1. Subjects with differentiated thyroid cancer split in subgroups based on thyroid peroxidase antibodies titers.

	DTC, n/total (%)	OR vs. Non-AITD 95% CI P value	OR among subgroups 95% CI P value
Non-AITD	600/1851 (32.4%)	n/a	n/a
TPO+ HT	48/129 37.2%)	1.24 0.85-1.79 0.286	0.43 (0.27-0.69) 0.0006
TPO- HT	96/166 (57.8%)	2.86 2.07-3.95 <0.0001	
TPO+ GRAVES	4/77 (5.2%)	0.11 0.04-0.31 <0.0001	0.27 (0.08 - 0.90) 0.043
TPO- GRAVES	10/59 (16.9%)	0.43 0.21-0.85 0.011	
TPO+ Euthyroid-HT	28/60 (46.7%)	1.82 1.09-3.06 0.025	0.44 (0.20-0.95) 0.038
TPO- Euthyroid-HT	34/51 (66.7%)	4.17 2.31-7.53 <0.0001	
TPO+ Hypothyroid-HT	20/69 (29.0%)	0.85 0.50-1.45 0.602	0.20 (0.09-0.45) <0.0001
TPO- Hypothyroid-HT	34/51 (66.7%)	4.17 2.31-7.53 <0.0001	

Odds ratios for the presence of differentiated thyroid cancer are estimated between subgroups of subjects with autoimmune thyroid diseases and subjects without autoimmune thyroid disease. The odds ratios for differentiated thyroid cancer are estimated between different subgroups as well.

Abbreviations: DTC: Differentiated Thyroid Cancer; OR: Odds ratio; Non-AITD: Subjects with pathology not consistent with any autoimmune thyroid disease; HT: Hashimoto's thyroiditis; Euthyroid-HT: subjects with Hashimoto's thyroiditis by pathology, with normal serum TSH without treatment with levothyroxine; Hypothyroid-HT: subjects with Hashimoto's thyroiditis by pathology, on treatment with levothyroxine preoperatively, due to a history of elevated TSH; GRAVES: subjects with Graves disease diagnosed by pathology; TPO+: subjects with thyroid peroxidase antibodies titers > 100IU/L; TPO-: subjects with thyroid peroxidase antibodies titers < 100IU/L.
